# Direct constraint control for EM-based miniaturization of microwave passives

**DOI:** 10.1038/s41598-022-17661-7

**Published:** 2022-08-03

**Authors:** Slawomir Koziel, Anna Pietrenko-Dabrowska

**Affiliations:** 1 Engineering Optimization & Modeling Center, Reykjavik University, 102 Reykjavik, Iceland; 2 Faculty of Electronics, Telecommunications and Informatics, Gdansk University of Technology, 80-233 Gdansk, Poland

**Keywords:** Electrical and electronic engineering, Computational science

## Abstract

Handling constraints imposed on physical dimensions of microwave circuits has become an important design consideration over the recent years. It is primarily fostered by the needs of emerging application areas such as 5G mobile communications, internet of things, or wearable/implantable devices. The size of conventional passive components is determined by the guided wavelength, and its reduction requires topological modifications, e.g., transmission line folding, or utilization of compact cells capitalizing on the slow-wave phenomenon. The resulting miniaturized structures are geometrically complex and typically exhibit strong cross coupling effects, which cannot be adequately accounted for by analytical or equivalent network models. Consequently, electromagnetic (EM)-driven parameter tuning is necessary, which is computationally expensive. When the primary objective is size reduction, the optimization task becomes far more challenging due to the presence of constraints related to electrical performance figures (bandwidth, power split ratio, etc.), which are all costly to evaluate. A popular solution approach is to utilize penalty functions. Therein, possible violations of constraints degrade the primary objective, thereby enforcing their satisfaction. Yet, the appropriate setup of penalty coefficients is a non-trivial problem by itself, and is often associated to extra computational expenses. In this work, we propose an explicit approach to constraint handling, which is combined with the trust-region gradient-search procedure. In our technique, the decision about the adjustment of the search radius is determined based on the reliability of rendering the feasible region boundary by linear approximation models of the constraints. Comprehensive numerical experiments conducted using three miniaturized coupler structures demonstrate superiority of the presented method over the penalty function paradigm. Apart from the efficacy, its appealing features include algorithmic simplicity, and no need for tailoring the procedure for a particular circuit to be optimized.

## Introduction

One of the important considerations in the design of modern high-frequency circuits and systems is miniaturization. Small size has become a prerequisite for a growing number of application areas that include mobile communications^1^, wearable^2^ and implantable devices^3^, internet of things^4^, medical imaging^5^, or energy harvesting^6^. Physical dimensions of conventional microwave passive components are related to the guided wavelength, which make them unsuitable for space-limited applications, except for structures implemented on high-permittivity substrates. In the context of circuit architecture, size reduction can be achieved by various means, including transmission line (TL) folding^7,8^, replacement of conventional TLs by compact microstrip resonant cells (CMRCs)^9^ capitalizing on slow-wave phenomenon^10^, as well as the employment of various geometrical modifications (e.g., defected ground structures^11^, slots^12^, stubs^13^, shorting pins^14^, substrate integrated waveguides^15^, etc.). The aforementioned techniques generally lead to complex, and often densely arranged layouts. The presence of electromagnetic (EM) cross-coupling effects within these structures makes the traditional characterization methods (analytical or equivalent network models) inadequate. Instead, reliable evaluation of miniaturized circuit has to rely on full-wave EM simulation tools.

Appropriate selection of the circuit architecture is only the first step of rendering a high-performance design. In order to achieve the smallest possible size while satisfying requirements imposed on the electrical parameters (allocation of operating frequencies, bandwidth, power split ratio, phase response), geometry parameters of the circuit have to be carefully tuned. Given multi-dimensional parameter spaces along with the necessity of handling several objectives and constraints, the parameter adjustment process needs to resort to rigorous numerical optimization algorithms^16,17^. At the same time, EM-driven optimization is computationally expensive: even local (e.g., gradient-based^18^ or derivative-free^19^) procedures may require many dozens of EM analyses, whereas tasks such as global search^20^, multi-objective design^21^, or uncertainty quantification^22^, are far costlier. Not surprisingly, the literature is replete with acceleration methods^23–27^. These include utilization of adjoint sensitivities^28^ or sparse Jacobian updates^29^ to expedite gradient-based procedures, the employment of dedicated solvers^30^, and, more and more popular, surrogate-based procedures^31^. The latter may employ data-driven (or approximation-based)^32,33^, and physics-based metamodels^34^, but also machine learning frameworks^35^. The latter are often combined with sequential sampling methodologies^36^ for iterative construction and refinement of the models. Some of popular approximation-based modelling methods in the context of EM-driven optimization include kriging^37^, Gaussian process regression^38^, neural networks in many variations (e.g.,^39–41^), support vector machines^42^, polynomial chaos expansion^43^. Physics-based metamodels are most often constructed using space mapping^44^, and response correction methods (e.g., adaptive response scaling^45^, manifold mapping^46^, etc.).

When it comes to EM-driven size reduction, a potentially high-cost of the process is only one of the challenges. The major issue is to control the constraints. As circuit miniaturization is generally detrimental to electrical performance figures, any practical design has to be a trade-off between achieving a possibly compact size and fulfilment of specifications imposed on the circuit characteristics. The latter are often expressed in terms of acceptance levels for return loss, bandwidth, power split, etc., over the frequency bands of interest. In mathematical terms, these conditions are essentially constraints. Their evaluation is computationally-heavy due to the involvement of EM analysis. Consequently, straightforward constraint handling is inconvenient. A widely used alternative is to incorporate penalty functions^47^, in which the main objective (size reduction) is supplemented with a linear combination of appropriately quantified constraint violations^48^. The advantage of this approach is problem reformulation, so that it becomes a formally unconstrained endeavor. Yet, the efficacy of optimization dependent on the setup of the proportionality factors of the aforementioned linear combination (referred to as penalty coefficients). Tailoring their values to a specific structure is non-trivial and typically requires execution of test runs, contributing to the overall computational cost of the process.

This paper discusses a novel methodology for simulation-driven miniaturization of microwave passive components. Our approach employs explicit handling of design constraints, which are approximated—in any given iteration of the optimization process—by their linear approximation models. The quality of this approximation, in particular the predictions concerning solution feasibility, are verified upon generating a new solution, and used to govern the decision-making process that controls the search radius within the trust-region procedure being the main optimization algorithm. The decision-making factors include the feasibility status of the current design, as well as the amount of constraint violation improvement (of the lack thereof). Furthermore, the tolerance levels for constraint violations are gradually tightened in the course of the optimization process, governed by its convergence indicators. The proposed constraint handling method is simple to implement and does not require any setup of control parameters (as opposed to penalty coefficients within the penalty function approach). It is validated using three structures of miniaturized rat-race and branch-line couplers with the constraints imposed on the circuit bandwidth and power split ratio. The obtained results are benchmarked against the penalty function techniques. We demonstrate that the presented procedure allows for a precise control over constraints, as well as for achieving competitive miniaturization rates. Perhaps its most appealing feature is that it does not have to be tuned to any specific circuit at hand.

## Miniaturization of microwave passives with direct constraint control

Here, we introduce the procedure for miniaturization of microwave components proposed in this work. “Simulation-based size reduction: problem formulation” provides the formulation of the miniaturization task. In “Explicit constraint handling: the concept”, we discuss the concept of direct control of CPU-heavy constraints within trust-region gradient-based algorithm. The technical details of controlling the tolerance levels for constraint violation as well as decision-making process that adjusts search radius are elaborated on in “Explicit constraint handling: constraint-related gain ratios”. Finally, “Explicit constraint handling: optimization algorithm” summarizes the entire procedure.

### Simulation-based size reduction: problem formulation

We denote by ***x*** = [*x*_1_ ⋯ *x*_*n*_]^*T*^ a vector of adjustable parameters of the circuit under design. For passive components, these are normally geometry parameters (circuit dimensions). Let *A*(***x***) be the circuit size, e.g., its footprint area. The objective is to reduce the size as much as possible, i.e., to solve

1 $${\varvec{x}}^{*} = \arg \mathop {\min }\limits_{{\varvec{x}}} U({\varvec{x}})$$

where x^*^ is the optimum parameter vector to be identified, whereas *U*(***x***) is the objective function. In the case of miniaturization, we have *U*(***x***) = *A*(***x***). The problem (Eq. 1) is subject to constraint, which can be of

2 $$\gamma_{k} ({\varvec{x}}) \le 0,\;\;\;k = 1,...,n_{\gamma }$$

or equality type

3 $$\eta_{k} ({\varvec{x}}) = 0,\;\;\;k = 1,...,n_{\eta }$$

In this work, we will only consider inequality constraints, which are the most common. Also, an equality constraint *η*_*k*_(***x***) = 0 can be represented in an inequality form as |*η*_*k*_(***x***)|≤ 0.

Let us consider an example of a microwave coupler, which is to be miniaturized while satisfying the following conditions.

- The power split ratio |*S*_31_(***x***,*f*) − *S*_21_(***x***,*f*)| is zero at *f* = *f*_0_ (the operating frequency);
- The matching and isolation characteristics are supposed to satisfy |*S*_11_(***x***,*f*)| ≤ − 20 dB, and |*S*_41_(***x***,*f*)| ≤ − 20 dB for *f* ∈ *F*, where *F* is a frequency range of interest (intended circuit bandwidth).

These conditions can be formulated as constraints *γ*_1_(***x***) ≤ 0 and *γ*_2_(***x***) ≤ 0, with *γ*_1_(***x***) =||*S*_31_(***x***,*f*) − *S*_21_(***x***,*f*)||, and *γ*_2_(***x***) = max{*f* ∈ *F*: max{|*S*_11_(***x***,*f*)|, |*S*_41_(***x***,*f*)|}} + 20.

Thus, for the exemplary coupler, we may formulate the miniaturization task as

4 $${\varvec{x}}^{*} = \arg \mathop {\min }\limits_{{\varvec{x}}} A({\varvec{x}})$$

subject to constraints

5 $$\left\| {S_{31} \left( {x,f} \right) - S_{21} \left( {x,f} \right)} \right\| \le 0$$

6 $$\max \left\{ {f \in F:\max \left\{ {S_{11} \left( {x,f_{0} } \right),S_{41} \left( {x,f_{0} } \right)} \right\}} \right\} + 20 \le 0$$

For the sake of illustration, let us consider another example of an optimization task, which is but oriented towards improving selected electrical performance figures rather than size reduction. Assume that the goal is to minimize the maximum reflection within the frequency range of interest of an impedance matching transformer. In this case, the merit function is defined as

7 $$U\left( {\varvec{x}} \right) = \max \left\{ {f \in F:\max \left\{ {S_{11} \left( {x,f_{0} } \right)} \right\}} \right\}$$

In Eq. (7), |*S*_11_(***x***,*f*)| stands for the circuit reflection, whereas *F* refers to the frequency range of interest.

### Explicit constraint handling: the concept

Evaluation of constraints imposed on electrical characteristics of microwave components is computationally expensive: their values are obtained by post-processing EM simulation data. This is troublesome from the point of view of numerical optimization procedures, as local methods typically require constraint gradients. Unless adjoint sensitivities are available^49^, estimation of these requires finite differentiation, and the constraints may not be differentiable due to their very formulation as minimax functions (cf. “Simulation-based size reduction: problem formulation”). Also, EM simulation results may contain a certain level of numerical noise, being a result of adaptive meshing techniques, or specific termination criteria used by the EM solvers. As mentioned before, a common mitigation method is a penalty function approach^47^, where the cost function is defined through aggregation of the main objective (size reduction) and contributions from constraint violations, appropriately scaled using weighting factors (penalty coefficients). Although conceptually attractive, optimum setup of the coefficient values is generally an intricate task, often associated with preparatory optimization runs.

This paper offers an alternative approach to constraint handling, which is an explicit method. It employs linear approximation models of the system response, therefore, a natural choice for the underlying optimization algorithm is the trust-region (TR) framework^50^. The standard TR procedure yields a series of approximate solutions ***x***^(*i*)^, *i* = 0, 1, …, that converge to x^*^. The new vector ***x***^(*i*+1)^ is obtained by solving

8 $${\varvec{x}}^{(i + 1)} = \arg \mathop {\min }\limits_{{{\varvec{x}};\;||{\varvec{x}} - {\varvec{x}}^{(i)} || \le \delta^{(i)} }} U_{L}^{(i)} ({\varvec{x}})$$

where *U*_*L*_^(*i*)^ is a first-order Taylor model of the scalar merit function *U*. The solution to Eq. (8) is restricted to the vicinity of ***x***^(*i*)^ determined by the size parameter *δ*^(*i*)^. Additionally, we have inequality constraints of the form of Eq. (2); equality constraints are not considered for the sake of simplicity, cf. “Simulation-based size reduction: problem formulation”. The TR radius *δ*^(*i*)^ is adaptively adjusted using the standard TR rules (e.g.^50^).

The problem (Eq. 8) is solved using Matlab’s *fmincon* algorithm, which implements the Sequential Quadratic Programming (SQP) procedure, one of the state-of-the-art procedures for constrained continuous optimization. The SQP procedure directly handles geometry constraints (here, the TR condition ||***x*** − ***x***^(*i*)^|| ≤ *δ*^(*i*)^), whereas the constraints related to electrical performance figures are controlled using the explicit approach being the subject of this paper. The details are explained in the remaining part of this section.

If size reduction is of interest, the evaluation of the merit function incurs negligible costs: the structure size can be obtained directly from the system geometry parameters, i.e., the vector ***x***. On the other hand, maintaining solution feasibility becomes problematic due to expensive constraints. In this work, constraint control is achieved by the incorporation of linearized models γ_*L.k*_ of the constraints γ_*k*_, *k* = 1, …, *n*_γ_, and adaptive adjustment of the trust-region size parameter *δ*^(*i*)^. The decision-making process governing the latter involves quantification of the reliability of γ_*L.k*_ in predicting the feasibility status in the course of the optimization process.

In the following, we will denote as ***r***(***x***) the vector of EM-simulated outputs (e.g., *S*-parameters) of the circuit of interest. A first-order Taylor model ***r***_***L***_^(*i*)^(***x***) of the response ***r***(***x***), established at the design ***x***^(*i*)^, is defined

9 $${\varvec{r}}_{{\varvec{L}}}^{(i)} ({\varvec{x}}) = {\varvec{r}}({\varvec{x}}^{(i)} ) + {\varvec{J}}({\varvec{x}}^{(i)} ) \cdot ({\varvec{x}} - {\varvec{x}}^{(i)} )$$

where the Jacobian matrix of the component response at the design ***x***^(*i*)^ is denoted as ***J***(***x***^(*i*)^). In most cases, it is estimated by means of finite differentiation. For the purpose of subsequent considerations, we will explicitly indicate that the constraints are functions of the circuit characteristics, i.e., we have *γ*_*k*_ = *γ*_*k*_(***r***(***x***)). Then, the linear model *γ*_*L.k*_ is defined as

10 $$\gamma_{L.k} ({\varvec{x}}) = \gamma_{k} ({\varvec{r}}_{{\varvec{L}}}^{(i)} ({\varvec{x}}))$$

When solving the trust-region sub-problem (Eq. 2), the exact constraints *γ*_*k*_(***r***(***x***)) will be replaced by their linearized versions (Eq. 10). The accuracy of representing *γ*_*k*_ by *γ*_*L.k*_ depends on a particular location in the parameter space and on the trust-region size parameter *δ*^(*i*)^, which is because ||*γ*_*k*_(***r***(***x***)) − *γ*_*L.k*_(***x***)|| is proportional to ||***x*** − ***x***^(*i*)^||^2^ (for sufficiently small design relocations). Consequently, a proper updating procedure for *δ*^(*i*)^ is essential. In particular, maintaining small values of the TR radius improves the alignment between *γ*_*L.k*_(***x***) and *γ*_*k*_(*r*(***x***)), whereas increasing it allows for increased-size steps in the design space while solving (Eq. 8). The adjustment of *δ*^(*i*)^ should take into account the solution feasibility predictions according to *γ*_*L.k*_(***x***), but also the actual feasibility status (as verified by EM simulation). At the generic level, the adaptation scheme is arranged the same way as for conventional TR algorithms^50^, i.e.,

11 $$\delta^{(i + 1)} = \left\{ {\begin{array}{*{20}l} {m_{inc\,} \delta^{(i)} } & {{\text{if}}\;\theta \ge \theta_{inc} } \\ {\delta^{(i)} } & {{\text{if}}\;\theta_{dec} \le \theta < \theta_{inc} } \\ {{{\delta^{(i)} } \mathord{\left/ {\vphantom {{\delta^{(i)} } {m_{dec} }}} \right. \kern-\nulldelimiterspace} {m_{dec} }}} & {{\text{if}}\;\theta < \theta_{dec} } \\ \end{array} } \right.$$

In Eq. (11), the coefficients *m*_*inc*_ and *m*_*dec*_ are used for incrementing and decrementing the TR region size, respectively, whereas *θ*_*inc*_ and *θ*_*dec*_ represent appropriate threshold. In our approach, we employ their typical values, i.e., we have *m*_*inc*_ = 2 and *m*_*inc*_ = 3, as well as *θ*_*inc*_ = 0.75 and *θ*_*dec*_ = 0.25^50^. As mentioned earlier, these are the typical values used in the TR algorithms. According to classical theory (e.g.^50^), the specific values of coefficients are not critical for the algorithm operation.

However, the decision-making factor *θ* in Eq. (11) is the gain ratio pertinent to the constraints. It compares the actual alteration of constraint violations to those predicted by the linear model for subsequent iterations (specifically, the (*i* + 1)th versus the *i*th one). The modification coefficients as well as the thresholds in Eq. (11) mimic the conventional rules of the TR frameworks (cf.^51^).

In the case of multiple constraints, the coefficient *ρ* is generalized to account for the worst-case situation over the entire set *g*_*k*_, *k* = 1, …, *n*_*g*_. We have

12 $$\theta = \min \{ \theta_{1} ,...,\theta_{{n_{\gamma } }} \}$$

Definition of the factors *θ*_*k*_ for each *γ*_*k*_(***x***), *k* = 1, …, *n*_*γ*_, is pivotal to the successful operation of the proposed optimization procedure. It will be discussed at length in the next section.

### Explicit constraint handling: constraint-related gain ratios

This section elaborates on the definition and evaluation of the constraint-based gain ratios *θ*_*k*_, utilized to control the trust region size as discussed in “Explicit constraint handling: the concept”. In the following, we will denote by *Γ*_*k*_^(*i*)^ the threshold for accepting the violation of *γ*_*k*_(***x***) at the iteration *i*. The threshold is iteration dependent for the reasons explained at the end of the section. At this point, we will outline the rules for computing the ratios *θ*_*k*_ utilized in decision-making process that governs the search radius adjustments:

- Rule 1: If *γ*_*k*_(***r***(***x***^(*i*)^)) > *Γ*_*k*_^(*i*)^, i.e., the constraint violation before executing the (*i* + 1)th iteration exceeds the acceptance threshold, then
  13 $$\theta_{k} = \frac{{\gamma_{k} ({\varvec{r}}({\varvec{x}}^{(i + 1)} )) - \gamma_{k} ({\varvec{r}}({\varvec{x}}^{(i)} ))}}{{\gamma_{L.k} ({\varvec{x}}^{(i + 1)} ) - \gamma_{L.k} ({\varvec{x}}^{(i)} )}}$$
- Rule 2: If *γ*_*k*_(***x***^(*i*)^) ≤ *Γ*_*k*_^(*i*)^, i.e., the constraint violation is at the acceptable level, then
  14 $$\theta_{k} = \frac{1}{2}\left[ {1 + {\text{sgn}} \left( {\gamma_{k} ({\varvec{r}}({\varvec{x}}^{(i)} )) - \gamma_{k} ({\varvec{r}}({\varvec{x}}^{(i + 1)} ))} \right)} \right]$$
- Rule 3: If *θ*_*k*_ < 0 (as computed using Eqs. (13) or (14)) but *γ*_*k*_(***r***(***x***^(*i*+1)^)) ≤ *Γ*_*k*_^(*i*)^, i.e., EM-evaluated constraint violation is acceptable, then the value of *θ*_*k*_ is overwritten to *θ*_*k*_ = 0.5.

**Figure 1:**
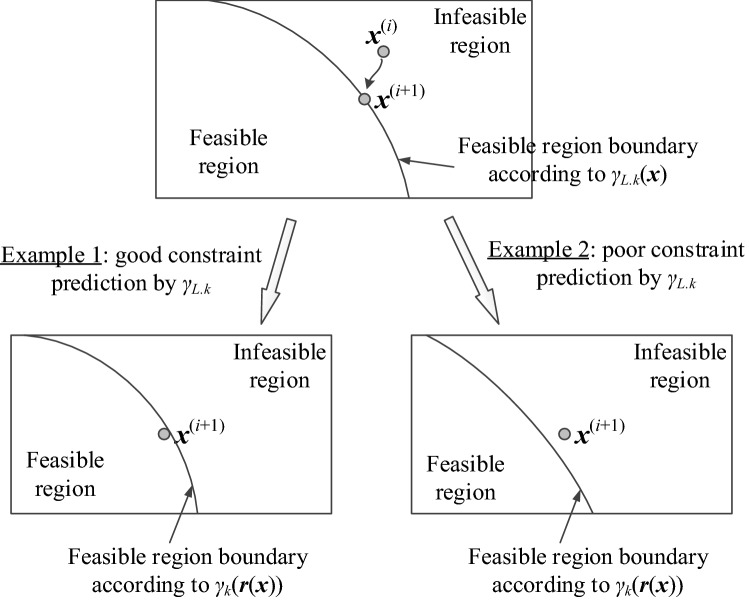
Prediction of design constraints by means of linear approximation model *γ*_*L.k*_ of (Eq. 10). The top picture illustrates relocation of the design from ***x***^(*i*)^ to ***x***^(*i*+1)^ obtained by solving (Eq. 8). In this example, ***x***^(*i*)^ is assumed feasible, whereas ***x***^(*i*+1)^ is allocated at the boundary of the feasible region according to the approximation model *γ*_*L.k*_. The bottom-left picture illustrates a case of satisfactory constraint prediction by *γ*_*L.k*_, i.e., the design ***x***^(*i*+1)^ is feasible according to the EM simulation data. The bottom-right picture shows a case of poor prediction: the design ***x***^(*i*+1)^ is infeasible according to the true constraint value evaluated through EM analysis. The latter will result in a reduction of the search region size *δ*^(*i*)^ in the next iteration (cf. Eqs. (13, 14)).

The above rules serve for two purposes. On the one hand, one needs to impose penalty on insufficient prediction accuracy of *γ*_*L.k*_ if the constraint violation is large (Rule 1), or the feasibility condition has not been improved in the case of minor constraint infringement (Rule 2). On the other hand, the conditions (Eqs. 13 and 14) are employed to promote sufficient prediction of *γ*_*k*_ by *γ*_*L.k*_ (cf. Eq. (13)), or relocation of the design towards the feasible region (cf. Eq. (14)). The role of Rule 3 is to overwrite the previous ones if the EM-evaluated constraint violation *γ*_*k*_(***r***(***x***^(*i*+1)^)) at the candidate design ***x***^(*i*+1)^ is within the acceptance threshold. Rule 3 has been introduced to prevent erratic operation for the designs residing in the vicinity of the feasible region boundary, in particular, near-zero constraint infringements (either positive or negative) in any given iteration. The graphical illustration of acceptable and insufficient evaluation of the constraint *γ*_*k*_ by the linearized model *γ*_*L.k*_ has been provided in Fig. 1.

In the remaining part of this section we discuss the acceptance thresholds *Γ*_*k*_^(*i*)^. At the early stages of the optimization process (far from convergence), it is advantageous to relax the acceptance thresholds for constraint violation in order to facilitate identification of small-size designs. However, when close to convergence, the thresholds should be tightened to ensure more precise control over constraints. In practice, this is realized by adjusting the threshold values based on the convergence status of the optimization process. Let *Γ*_*k.*max_ be a user-defined maximum violation level. Further, let *ε* be a small positive number determining the algorithm termination. In this work, the termination condition is formulated as ||***x***^(*i*+1)^ − ***x***^(*i*)^||< *ε* or *δ*^(*i*)^ < *ε*. For a given iteration *i*, let us define the convergence indicator

15 $$\psi^{(i)} = \max \left\{ {1,\frac{{\min \left\{ {||{\varvec{x}}^{(i + 1)} - {\varvec{x}}^{(i)} ||,\delta^{(i)} } \right\}}}{\varepsilon }} \right\}$$

Note that *ψ*^(*i*)^ is large when the optimization process is launched, and it is reduced to unity upon convergence. We also have the threshold

16 $$\Gamma_{k}^{(i + 1)} = \Gamma_{k.\max } \min \left\{ {1,\alpha \psi^{(i)} } \right\}$$

As *Γ*_*k*_^(*i*+1)^ is proportional to *ψ*^(*i*)^, it is initially equal to *Γ*_*k.*max_, and gradually diminished to *αΓ*_*k.*max_ upon convergence. Here, *α* is assumes small values greater than zero, e.g., 0.1 or 0.01. This value is not of key importance for the operation of the procedure.

### Explicit constraint handling: optimization algorithm

**Figure 2:**
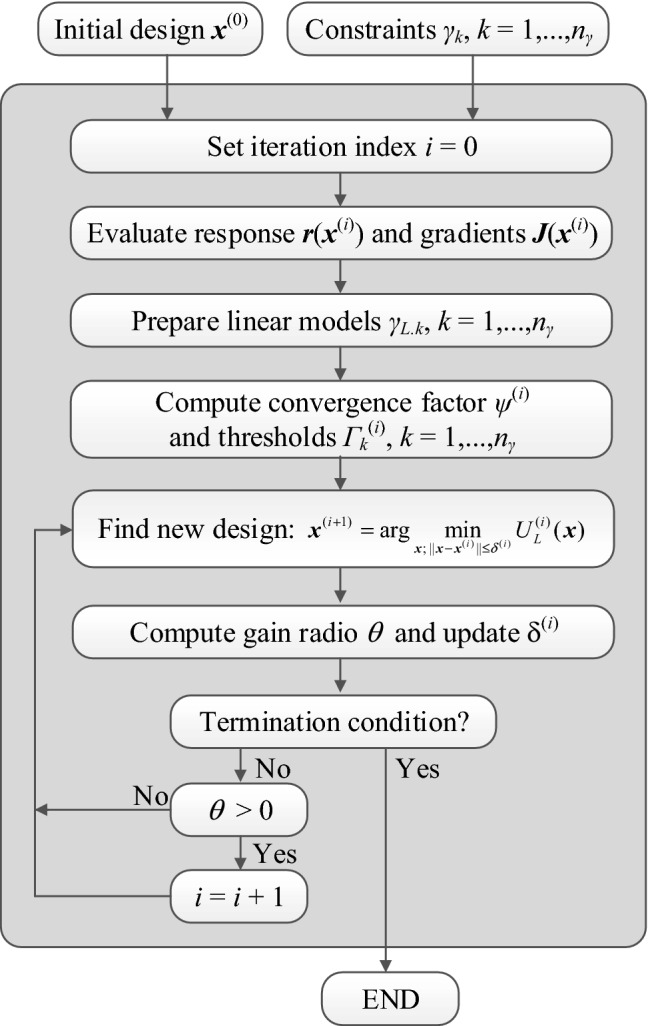
Flow diagram of the proposed size reduction algorithm with explicit constraint handling.

Figure 2 shows the operating flowchart of the proposed size reduction procedure with explicit handling of design constraints. Apart from the termination threshold, the algorithm only contains the following control parameters: the threshold *Γ*_*k*.max_ (maximum tolerance for constraint violation), and the scaling coefficient *α*. These parameters are not critical for the algorithm performance. As a matter of fact, we will keep these values fixed for all verification case studies considered in “Demonstration examples”. The acceptance of the candidate design ***x***^(*i*+1)^ produced by solving (Eq. 8) is based on the standard TR rules, i.e., it is accepted if the decision-making factor *θ* is positive, and rejected otherwise. In the latter case, the iteration is repeated with a reduced search region. Note that *θ* > 0 if either the violation of the constraint has been reduced to a sufficient extent, or the design was relocated to the feasible region.

## Demonstration examples

This section summarizes the results of numerical experiments conducted to validate and benchmark the proposed size reduction approach. Verification is based on three compact microstrip couplers, including a branch-line and two rat-race circuits. Our procedure is compared to optimization involving penalty function approach in several variations featuring different setups of penalty coefficients. The performance figures of interest include the obtained circuit size, as well as the accuracy of controlling the constraints, related to the circuit bandwidth and the power split ratio.

### Verification circuits

**Figure 3:**
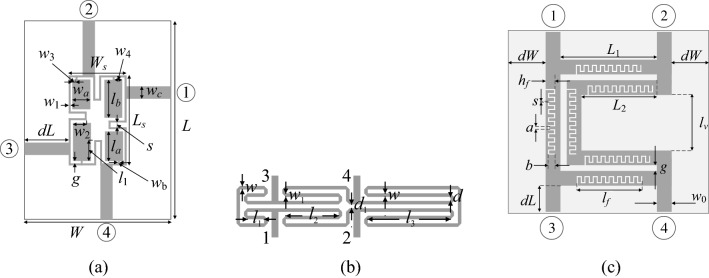
Passive microstrip components utilized for verification of the proposed optimization procedure: (**a**) compact branch-line coupler (Circuit I)^52^, (**b**) rat-race coupler with folder transmission lines (Circuit II)^53^, (**c**) rat-race coupler with defected microstrip structure (Circuit III)^54^.

The considered circuits have been shown in Fig. 3. Their computational models are implemented in CST Microwave Studio and evaluated using the time-domain solver. In all cases, the main objective is reduction of the circuit footprint area. There are two constraints imposed on the circuit *S*-parameters: *γ*_1_(***x***) =||*S*_31_(***x***,*f*_0_) − *S*_21_(***x***,*f*_0_)|| − 0.1 dB, and *γ*_2_(***x***) = max{*f* ∈ *F*: max{|*S*_11_(***x***,*f*)|, |*S*_41_(***x***,*f*)|}} + 20 dB, where *f*_0_ is the center frequency, and *F* is the intended circuit bandwidth. Two scenarios are considered with different choice of bandwidth for each circuit.

The first constraint enforces equal power split ratio within 0.1 dB tolerance, whereas the second ensures that the circuit impedance matching and port isolation are better or equal − 20 dB within the operating band. Table 1 provides essential data about all three structures.

**Table 1:** Benchmark microwave components.

	Case study		
	Circuit I	Circuit II	Circuit III
Substrate	AD300 (*ε*_*r*_ = 2.97, *h* = 0.76 mm)	RO4003 (*ε*_*r*_ = 3.38, *h* = 0.762 mm)	FR4 (*ε*_*r*_ = 4.4, *h* = 1.55 mm)
Design parameters	***x*** = [*g l*_1*r*_* l*_*a*_* l*_*b*_* w*_1_ *w*_2*r*_* w*_3*r*_* w*_4*r*_* w*_*a*_* w*_*b*_]^*T*^	***x*** = [*l*_1_ *l*_2_ *l*_3_ *d w w*_1_]^*T*^	***x*** = [*L*_1_ *b*_*r*_* g h*_*fr*_* s l*_*fr*_]^*T*^
Other parameters	*L* = 2*dL* + *L*_*s*_, *L*_*s*_ = 4*w*_1_ + 4* g* + s + *l*_*a*_ + *l*_*b*_, *W* = 2*dL* + *W*_*s*_, *W*_*s*_ = 4*w*_1_ + 4* g* + *s* + 2*w*_*a*_, *l*_1_ = *l*_*b*_*l*_1*r*_, w_2_ = *w*_*a*_*w*_2*r*_, *w*_3_ = *w*_3*r*_*w*_*a*_, and *w*_4_ = *w*_4*r*_*w*_*a*_, *w*_*c*_ = 1.9 mm	*d*_1_ = *d *+ |*w* − *w*_1_|, *d* = 1.0, *w*_0_ = 1.7, and *l*_0_ = 15 mm	*L*_2_ = *L*_1_ − *g* − *w*_0_*, a* = (*l*_*f*_ − 17*s*)/16, *b* = (*h*_*f*_ − *s*)*b*_*r*_, *l*_*f*_ = *L*_2_ *l*_*fr*_, *l*_*v*_ = *L*_1_ − 2*g* − *2w*_0_, and *h*_*f*_ = *s* + (*w*_0_ − *s*)*h*_*fr*_; *dW* = *dL* = 10 mm
Operating parameters (design scenario I)	*f*_0_ = 1.5 GHz *F* = [1.45 1.55] GHz	*f*_0_ = 1.0 GHz *F* = [0.9 1.1] GHz	*f*_0_ = 1.2 GHz *F* = [1.15 1.25] GHz
Operating parameters (design scenario II)	*f*_0_ = 1.5 GHz *F* = [1.47 1.53] GHz	*f*_0_ = 1.0 GHz *F* = [0.95 1.05] GHz	*f*_0_ = 1.2 GHz *F* = [1.18 1.22] GHz
Initial design^a^	***x***^(0)^ = [0.45 0.69 6.25 10.32 0.96 0.39 0.14 0.57 4.62 0.60]^*T*^	***x***^(0)^ = [5.27 13.33 21.51 0.96 0.89 0.90]^*T*^	***x***^(0)^ = [31.79 0.67 2.12 0.80 0.49 0.33]^*T*^

^a^Initial design obtained by optimizing the circuit for best matching/isolation within the frequency range *F*, under equal-power-split constraint.

### Experimental setup and results

The proposed optimization procedure has been applied to the circuits of Fig. 3. In each case, the initial design (the last row of Table 1) was obtained by optimizing the respective circuits to improve the matching and isolation within the operating bandwidth F, subject to equal power split constraint. This means, in particular, that the starting points are feasible from the point of view of both constraints *γ*_1_ and *γ*_2_ (cf. “Verification circuits”). The termination threshold is set to *ε* = 10^−3^, the acceptance threshold are chosen to be *Γ*_1.max_ = 1 dB, *Γ*_2.max_ = 0.3 dB, and *α* = 0.1.

The results are compared to the algorithm employing the penalty function approach. Therein, the objective function is defined as

17 $$U({\varvec{x}}) = A({\varvec{x}}) + \beta_{1} c_{1} ({\varvec{x}})^{2} + \beta_{2} c_{2} ({\varvec{x}})^{{}}$$

where the penalty functions *c*_1_ and *c*_2_ measure relative constraint violations, i.e., we have

18 $$c_{1} ({\varvec{x}}) = \max \left\{ {0,\frac{{\gamma_{1} ({\varvec{x}}) + 0.1}}{0.1}} \right\}\;{\text{and}}\;c_{2} ({\varvec{x}}) = \max \left\{ {0,\frac{{\gamma_{2} ({\varvec{x}}) + 20}}{20}} \right\}$$

The benchmark algorithm is run for all combinations of the penalty coefficients *β*_1_ ∈ {10, 100, 1000, 10,000}, *β*_2_ ∈ {10, 100, 1000, 10,000}. This is to illustrate the fact that optimum performance of the algorithm requires identification of the appropriate setup of the penalty terms, and sub-optimal setup leads to inferior constraint control or miniaturization rates.

**Figure 4:**
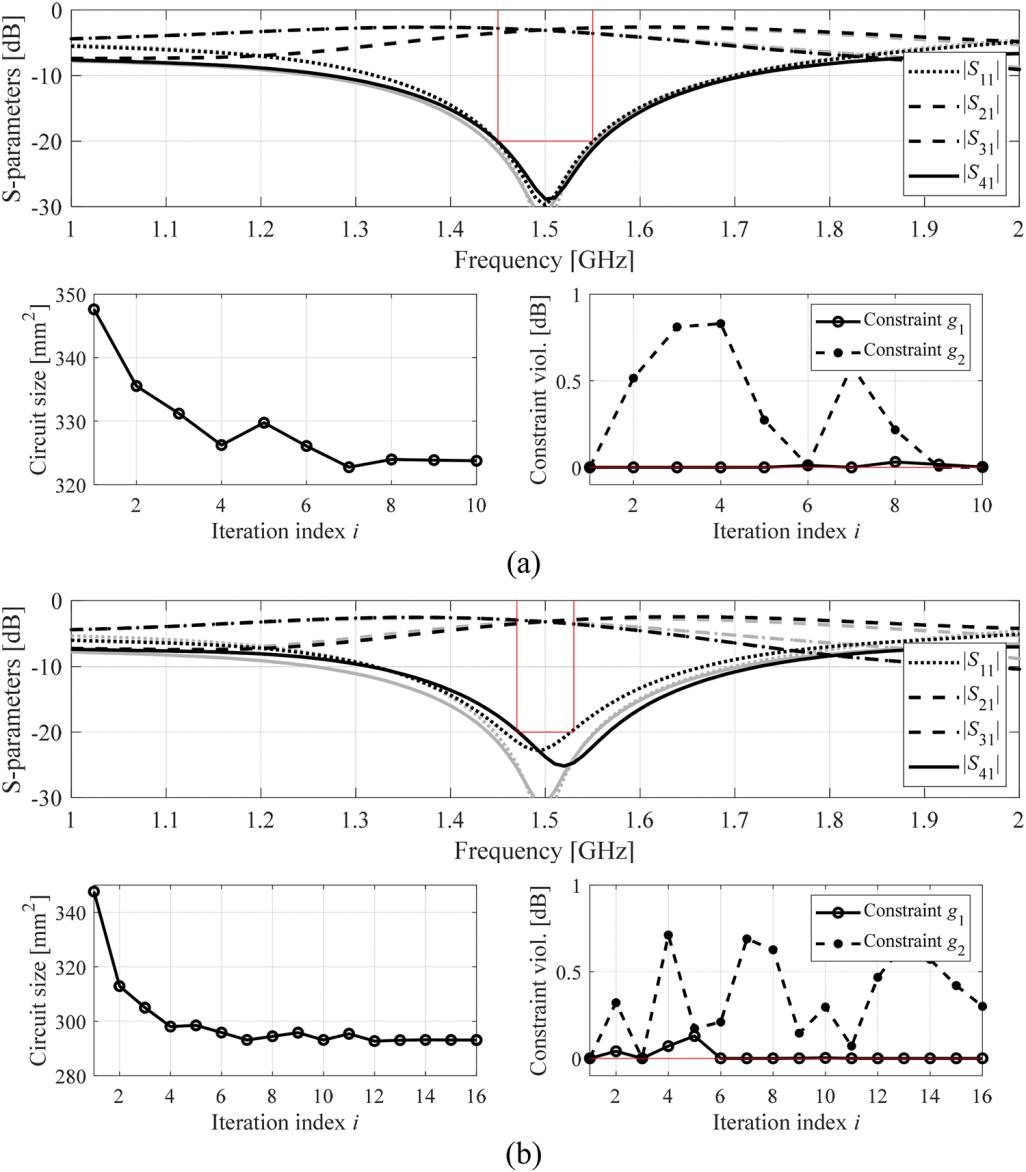
Initial (gray) and optimized (black) *S*-parameters of Circuit I. The vertical and horizontal lines mark the target operating bandwidth and the acceptance level for the matching |*S*_11_| and isolation |*S*_41_| responses. Also shown is the evolution of the circuit size and constraint violations (in case of feasibility, violations shown as zero): (**a**) design scenario I (bandwidth 1.45–1.55 GHz), (**b**) design scenario II (bandwidth 1.47–1.53 GHz).

**Figure 5:**
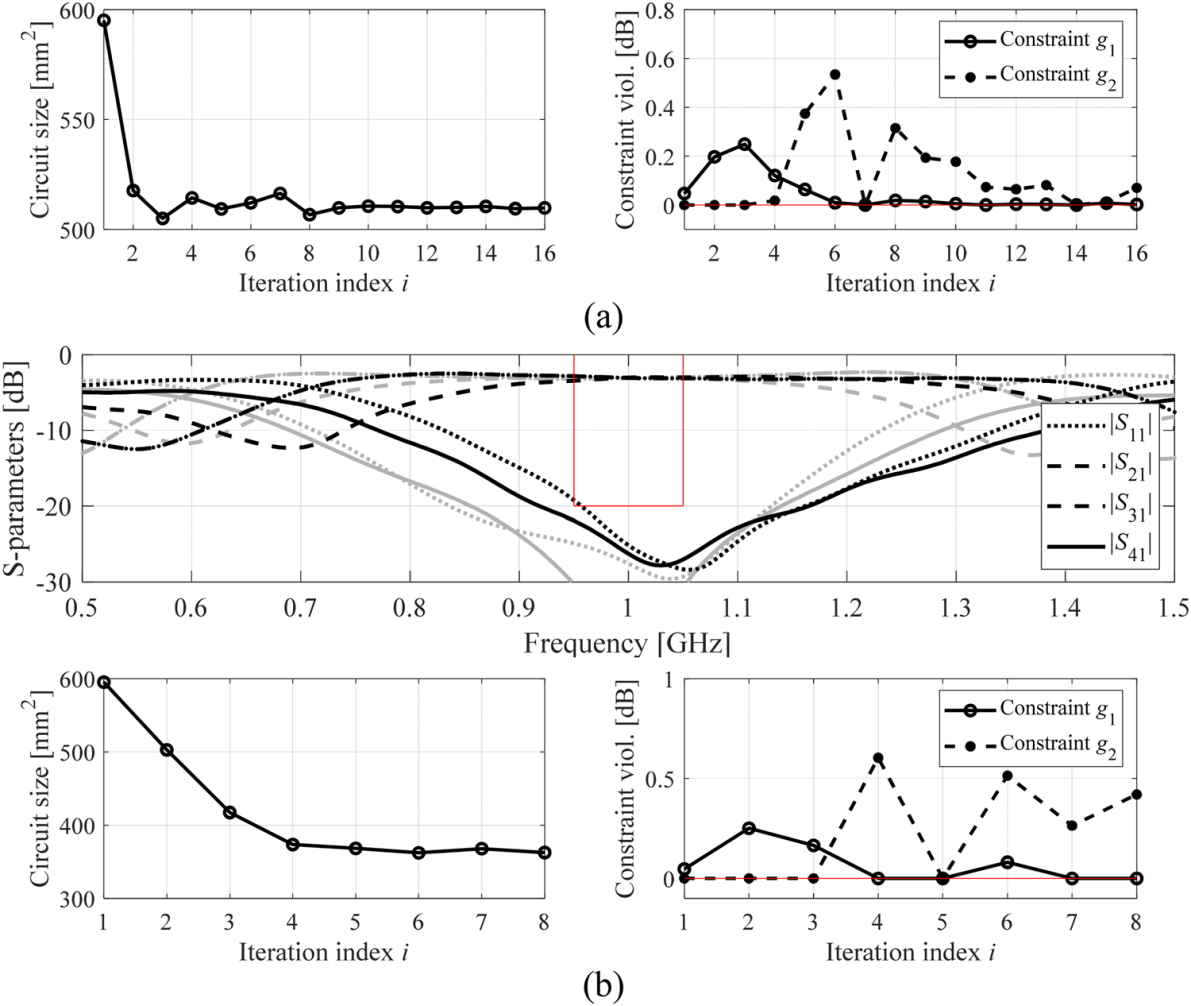
Initial (gray) and optimized (black) *S*-parameters of Circuit II. The vertical and horizontal lines mark the target operating bandwidth and the acceptance level for the matching |*S*_11_| and isolation |*S*_41_| responses. Also shown is the evolution of the circuit size and constraint violations (in case of feasibility, violations shown as zero): (**a**) design scenario I (bandwidth 0.9–1.1 GHz), (**b**) design scenario II (bandwidth 0.95–1.05 GHz).

**Figure 6:**
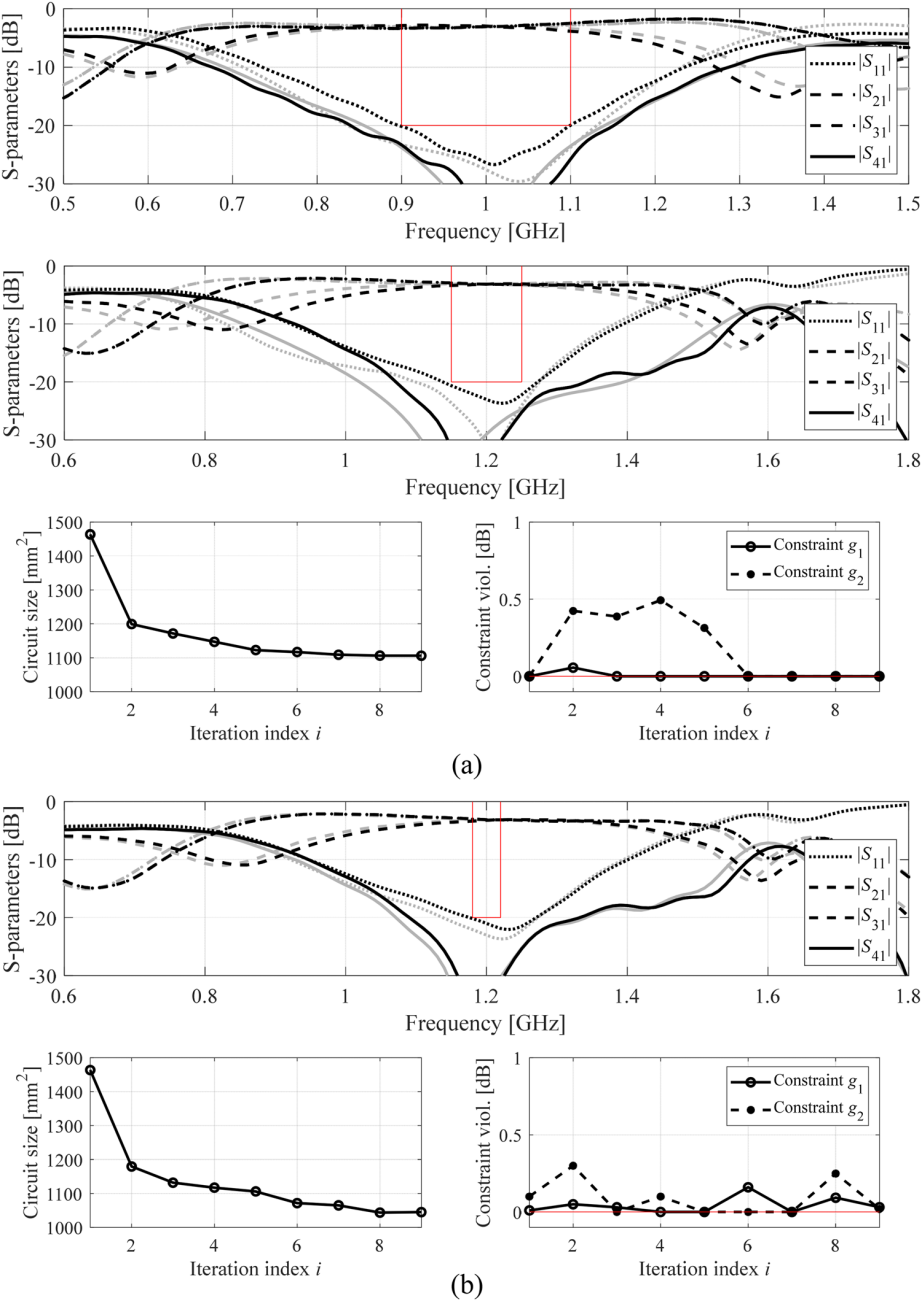
Initial (gray) and optimized (black) *S*-parameters of Circuit III. The vertical and horizontal lines mark the target operating bandwidth and the acceptance level for the matching |*S*_11_| and isolation |*S*_41_| responses. Also shown is the evolution of the circuit size and constraint violations (in case of feasibility, violations shown as zero): (**a**) design scenario I (bandwidth 1.15–1.25 GHz), (**b**) design scenario II (bandwidth 1.18–1.22 GHz).

The numerical results have been gathered in Tables 2, 3 and 4. These include the achieved footprint area of the respective circuits, as well as constraint violations at the final design. Figures 4, 5 and 6 show the circuit characteristics at the initial and the optimized designs for all circuits, along with the history of the circuit footprint area and violation of constraints during the optimization run.

**Table 2:** Optimization results for Circuit I.

Optimization approach		Performance parameters					
		Design scenario I (*F* = [1.45 1.55] GHz)			Design scenario II (*F* = [1.47 1.53] GHz)		
Method	Setup	Footprint area *A* (mm^2^)	Violation of constraint *γ*_1_ (dB)	Violation of constraint *γ*_2_ (dB)	Footprint area *A* (mm^2^)	Violation of constraint *γ*_1_ (dB)	Violation of constraint *γ*_2_ (dB)
Implicit constraint handling (penalty function approach)	*β*_1_ = 10^1^, *β*_2_ = 10^1^	241	0.03	6.8	264	0.07	3.5
	*β*_1_ = 10^1^, *β*_2_ = 10^2^	259	0.06	5.3	264	0.07	3.5
	*β*_1_ = 10^1^, *β*_2_ = 10^3^	301	− 0.01	1.9	272	0.02	2.1
	*β*_1_ = 10^1^, *β*_2_ = 10^4^	325	0.01	0.2	293	0.02	0.2
	*β*_1_ = 10^2^, *β*_2_ = 10^1^	247	− 0.05	6.6	264	0.07	3.5
	*β*_1_ = 10^2^, *β*_2_ = 10^2^	258	− 0.02	5.7	276	0.00	1.7
	*β*_1_ = 10^2^, *β*_2_ = 10^3^	318	0.01	1.0	292	− 0.01	0.5
	*β*_1_ = 10^2^, *β*_2_ = 10^4^	319	0.00	0.3	297	− 0.08	0.3
	*β*_1_ = 10^3^, *β*_2_ = 10^1^	247	− 0.04	7.1	333	− 0.00	0.5
	*β*_1_ = 10^3^, *β*_2_ = 10^2^	264	− 0.03	53	335	− 0.01	1.0
	*β*_1_ = 10^3^, *β*_2_ = 10^3^	318	− 0.01	1.3	322	− 0.02	− 1.1
	*β*_1_ = 10^3^, *β*_2_ = 10^4^	319	0.00	0.2	301	− 0.05	0.1
	*β*_1_ = 10^4^, *β*_2_ = 10^1^	242	0.00	6.9	323	− 0.00	0.5
	*β*_1_ = 10^4^, *β*_2_ = 10^2^	258	− 0.05	5.7	292	− 0.06	0.8
	*β*_1_ = 10^4^, *β*_2_ = 10^3^	310	− 0.03	1.4	325	− 0.00	0.0
	*β*_1_ = 10^4^, *β*_2_ = 10^4^	317	0.00	0.4	302	− 0.07	0.1
Explicit constraint handling (this work)		323	0.00	0.0	293	− 0.05	0.3

**Table 3:** Optimization results for Circuit II.

Optimization approach		Performance parameters					
		Design scenario I (*F* = [0.9 1.1] GHz)			Design scenario II (*F* = [0.95 1.05] GHz)		
Method	Setup	Footprint area *A* (mm^2^)	Violation of constraint *γ*_1_ (dB)	Violation of constraint *γ*_2_ (dB)	Footprint area *A* (mm^2^)	Violation of constraint *γ*_1_ (dB)	Violation of constraint *γ*_2_ (dB)
Implicit constraint handling (penalty function approach)	*β*_1_ = 10^1^, *β*_2_ = 10^1^	124	0.01	16.8	114	0.00	16.6
	*β*_1_ = 10^1^, *β*_2_ = 10^2^	104	0.02	17.0	90	0.00	17.6
	*β*_1_ = 10^1^, *β*_2_ = 10^3^	464	0.27	3.2	439	0.21	2.9
	*β*_1_ = 10^1^, *β*_2_ = 10^4^	508	0.17	− 0.1	364	− 0.09	0.2
	*β*_1_ = 10^2^, *β*_2_ = 10^1^	593	0.04	− 3.4	593	0.04	− 5.2
	*β*_1_ = 10^2^, *β*_2_ = 10^2^	593	0.04	− 3.4	593	0.04	− 5.2
	*β*_1_ = 10^2^, *β*_2_ = 10^3^	538	0.07	− 1.9	593	0.04	− 5.2
	*β*_1_ = 10^2^, *β*_2_ = 10^4^	593	0.04	− 3.4	593	0.04	− 5.2
	*β*_1_ = 10^3^, *β*_2_ = 10^1^	595	0.04	− 3.4	595	0.04	− 5.2
	*β*_1_ = 10^3^, *β*_2_ = 10^2^	595	0.04	− 3.4	595	0.04	− 5.2
	*β*_1_ = 10^3^, *β*_2_ = 10^3^	595	0.04	− 3.4	595	0.04	− 5.2
	*β*_1_ = 10^3^, *β*_2_ = 10^4^	595	0.04	− 3.4	595	0.04	− 5.2
	*β*_1_ = 10^4^, *β*_2_ = 10^1^	595	0.04	− 3.4	595	0.04	− 5.2
	*β*_1_ = 10^4^, *β*_2_ = 10^2^	595	0.04	− 3.4	595	0.04	− 5.2
	*β*_1_ = 10^4^, *β*_2_ = 10^3^	595	0.04	− 3.4	595	0.04	− 5.2
	*β*_1_ = 10^4^, *β*_2_ = 10^4^	595	0.04	− 3.4	595	0.04	− 5.2
Explicit constraint handling (this work)		510	0.00	0.1	363	− 0.03	0.4

**Table 4:** Optimization results for Circuit III.

Optimization approach		Performance parameters					
		Design scenario I (*F* = [1.15 1.25] GHz)			Design scenario II (*F* = [1.18 1.22] GHz)		
Method	Setup	Footprint area *A* (mm^2^)	Violation of constraint *γ*_1_ (dB)	Violation of constraint *γ*_2_ (dB)	Footprint area *A* (mm^2^)	Violation of constraint *γ*_1_ (dB)	Violation of constraint *γ*_2_ (dB)
Implicit constraint handling (penalty function approach)	*Z`β*_1_ = 10^1^, *β*_2_ = 10^1^	1067	0.17	0.7	1043	0.12	− 0.7
	*β*_1_ = 10^1^, *β*_2_ = 10^2^	681	0.01	10.4	679	0.00	9.4
	*β*_1_ = 10^1^, *β*_2_ = 10^3^	1063	− 0.03	0.1	1063	− 0.03	− 1.0
	*β*_1_ = 10^1^, *β*_2_ = 10^4^	1097	0.02	− 0.1	1097	0.02	− 1.2
	*β*_1_ = 10^2^, *β*_2_ = 10^1^	1120	0.04	0.6	1120	0.04	− 0.5
	*β*_1_ = 10^2^, *β*_2_ = 10^2^	1134	0.00	− 0.3	1134	0.00	− 1.7
	*β*_1_ = 10^2^, *β*_2_ = 10^3^	1133	0.00	0.1	1133	0.00	− 1.2
	*β*_1_ = 10^2^, *β*_2_ = 10^4^	1038	− 0.03	1.1	1038	− 0.03	0.0
	*β*_1_ = 10^3^, *β*_2_ = 10^1^	1165	− 0.05	− 0.3	1165	− 0.05	− 1.7
	*β*_1_ = 10^3^, *β*_2_ = 10^2^	1119	0.01	− 0.1	1119	0.01	− 1.3
	*β*_1_ = 10^3^, *β*_2_ = 10^3^	1152	− 0.06	− 0.3	1152	− 0.06	− 1.6
	*β*_1_ = 10^3^, *β*_2_ = 10^4^	1117	− 0.08	− 0.1	1047	− 0.08	− 1.7
	*β*_1_ = 10^4^, *β*_2_ = 10^1^	1218	0.00	− 0.0	1136	− 0.02	0.2
	*β*_1_ = 10^4^, *β*_2_ = 10^2^	1208	0.00	− 0.2	1132	0.01	− 2.1
	*β*_1_ = 10^4^, *β*_2_ = 10^3^	1152	0.00	− 0.5	1152	0.00	− 1.7
	*β*_1_ = 10^4^, *β*_2_ = 10^4^	1152	− 0.02	− 0.1	1134	0.00	− 2.2
Explicit constraint handling (this work)		1106	− 0.04	− 0.1	1045	0.01	− 0.1

### Discussion

The results presented in “Experimental setup and results” allow us to formulate a number of remarks concerning performance of the proposed optimization procedure with explicit handling of design constraints. Furthermore, our methodology can be conclusively compared with the benchmark methods employing the penalty function approach. The observations are as follows.

- The proposed algorithm performs consistently for all considered verification circuits. In particular, it enables a satisfactory control of both design constraints. There is no violation for power split ratio constraint observed, whereas the maximum violation for the matching/isolation constraint is only 0.5 dB for Circuit II (design scenario II); however, it should be noted that the acceptance threshold is − 20 dB.
- The performance of benchmark methods is highly dependent on the penalty coefficient setup. Among sixteen combination of parameters, only a few lead to satisfactory results in terms of both ensuring good miniaturization ratio and sufficiently precise constraint handling. For Circuit I we have about three of such ‘good’ setups, for Circuit II there is only one (per design scenario), whereas for Circuit III about three.
- For the particular setups ensuring good performance of the benchmark procedure, the obtained circuit sizes are comparable to those obtained by the proposed algorithm (which, on the other hand, does not require any setup or tailoring to the task at hand).
- In the case of Circuit II, for most combinations of penalty coefficients featuring *β*_1_ ≥ 10^2^, the optimization process becomes stuck at the early stages of the optimization process, leaving large feasibility margin for the second constraint. This is due to the fact that a large value of the first penalty coefficient along with a small margin for the power split ratio constraint (only 0.1 dB), makes the problem numerically challenging. More specifically, the objective function becomes highly nonlinear near the feasible region boundary, which hinders exploration of that region and leads to a premature convergence of the process. A similar effect can be observed for Circuit III, although it is less pronounced.
- The proposed algorithm turns out to be less prone to the aforementioned issues due to the adaptive adjustment of the acceptance thresholds governed by the convergence status of the algorithm (cf. Eqs. (15, 16)).
- The average costs of rendering the optimal designs by the proposed approach equal: 110, 80 and 55 full-wave simulations, for Circuit I, II and III, respectively. Whereas in the case of the benchmark procedure the corresponding costs are: 115, 57 and 45 EM simulations. Therefore, the proposed procedure is around 20% more expensive in terms of the number of EM analyses necessary for the algorithm to converge. Yet, given that in our approach there is virtually no need for tailoring the algorithm to render a satisfactory design meeting the design specifications, this additional cost seems to be justifiable. This is because tuning the optimization procedure to ensure its satisfactory operation normally entails additional computational expenses (e.g., for adjusting penalty coefficients in the case of implicit methods). Furthermore, the primary purpose of the presented technique was to improve the precision of controlling design constraints, and miniaturization rate, both of which have been conclusively demonstrated.

Given a large combined number of circuits, design scenarios, as well as penalty coefficient setups involved in this verification study, the observations summarized above should be categorized as conclusive. Overall, the performance of the presented procedure can be considered competitive over the benchmark (implicit) methods, both with respect to the accuracy of constraint handling and achievable miniaturization rates. The important advantages of the proposed algorithm include easy implementation and no need for adjusting any control parameters. The latter normally incurs extra computational expenses and may require a certain level of experience pertinent to optimization methods.

## Conclusion

The purpose of this work was to propose a novel procedure for simulation-based miniaturization of microwave passives. Our approach involves direct control of constraints imposed on electrical performance figures of the circuit under design. Linear approximation models of the constraint functions are employed to make predictions concerning solution feasibility. Appropriately quantified quality of these predictions is utilized in the decision-making process that controls the search region size within the trust region framework. Furthermore, the constraint violation tolerance thresholds are governed by the convergence indicators of the optimization process in order to foster more aggressive size reduction at the early stages of the optimization process. Comprehensive numerical verification involving three microstrip couplers and six design scenarios demonstrate superior performance of the proposed technique as compared to the benchmark methods employing a penalty function approach for implicit constraint handling. Its major advantages include competitive size reduction ratios, accuracy in controlling constraint violation levels, consistency of results obtained for a variety of problems, straightforward implementation, as well as no need for tailoring the procedure to handle a particular microwave structure. The last feature is particularly important in practical applications: tuning the optimization procedure to ensure satisfactory operation (e.g., setting up penalty coefficients for implicit methods) normally entails additional computational expenses and may require optimization-related knowhow lacking by many microwave engineers.

## Data Availability

The datasets generated during and/or analysed during the current study are available from the corresponding author on reasonable request. Contact person: anna.dabrowska@pg.edu.pl.

## References

[1] Khan MS, Iftikhar A, Shubair RM, Capobianco A, Braaten BD, Anagnostou DE (2020). Eight-element compact UWB-MIMO/diversity antenna with WLAN band rejection for 3G/4G/5G communications. IEEE Open J. Ant. Propag..

[2] Jiang ZH, Gregory MD, Werner DH (2016). Design and experimental investigation of a compact circularly polarized integrated filtering antenna for wearable biotelemetric devices. IEEE Trans. Biomed. Circ. Syst..

[3] Kracek J, Švanda M, Mazanek M, Machac J (2016). Implantable semi-active UHF RFID tag with inductive wireless power transfer. IEEE Ant. Wireless Propag. Lett..

[4] Zhang J, Yan S, Hu X, Vandenbosch GAE (2019). Dual-band dual-polarized wearable button array with miniaturized radiator. IEEE Trans. Biomed. Circ. Syst..

[5] Zhang H, Li M, Yang F, Xu S, Zhou H, Yang Y, Chen L (2020). A low-profile compact dual-band L-shape monopole antenna for microwave thorax monitoring. IEEE Ant. Wireless Propag. Lett..

[6] He Z, Liu C (2020). “A compact high-efficiency broadband rectifier with a wide dynamic range of input power for energy harvesting. IEEE Microwave Wireless Comp. Lett..

[7] Martinez L, Belenguer A, Boria VE, Borja AL (2019). Compact folded bandpass filter in empty substrate integrated coaxial line at S-Band. IEEE Microwave Wireless Comp. Lett..

[8] Hou ZJ, Yang Y, Zhu X, Li YC, Dutkiewicz E, Xue Q (2018). A compact and low-loss bandpass filter using self-coupled folded-line resonator with capacitive feeding technique. IEEE Electron Dev. Lett..

[9] Chen S, Guo M, Xu K, Zhao P, Dong L, Wang G (2018). A frequency synthesizer based microwave permittivity sensor using CMRC structure. IEEE Access.

[10] Qin W, Xue Q (2013). Elliptic response bandpass filter based on complementary CMRC. Electr. Lett..

[11] Sen S, Moyra T (2019). Compact microstrip low-pass filtering power divider with wide harmonic suppression. IET Microwaves Ant. Propag..

[12] Zhang W, Shen Z, Xu K, Shi J (2019). A compact wideband phase shifter using slotted substrate integrated waveguide. IEEE Microwave Wireless Comp. Lett..

[13] Wei F, Jay Guo Y, Qin P, WeiShi X (2015). Compact balanced dual- and tri-band bandpass filters based on stub loaded resonators. IEEE Microwave Wireless Comp. Lett..

[14] Yang D, Zhai H, Guo C, Li H (2020). A compact single-layer wideband microstrip antenna with filtering performance. IEEE Antennas Wireless Propag. Lett..

[15] Liu S, Xu F (2018). Compact multilayer half mode substrate integrated waveguide 3-dB coupler. IEEE Microwave Wireless Comp. Lett..

[16] Rayas-Sanchez JE, Koziel S, Bandler JW (2021). Advanced RF and microwave design optimization: A journey and a vision of future trends. IEEE J. Microwaves.

[17] Zhang F, Li J, Lu J, Xu C (2021). Optimization of circular waveguide microwave sensor for gas-solid two-phase flow parameters measurement. IEEE Sensors J..

[18] Feng F, Na W, Liu W, Yan S, Zhu L, Zhang Q-J (2020). Parallel gradient-based EM optimization for microwave components using adjoint- sensitivity-based neuro-transfer function surrogate. IEEE Trans. Microwave Theory Techn..

[19] Na W, Liu K, Cai H, Zhang W, Xie H, Jin D (2021). Efficient EM optimization exploiting parallel local sampling strategy and Bayesian optimization for microwave applications. IEEE Microwave Wireless Comp. Lett..

[20] Koziel S, Pietrenko-Dabrowska A, Al-Hasan M (2020). Improved-efficacy optimization of compact microwave passives by means of frequency-related regularization. IEEE Access.

[21] Güneş F, Uluslu A, Mahouti P (2020). Pareto optimal characterization of a microwave transistor. IEEE Access.

[22] Ochoa JS, Cangellaris AC (2013). Random-space dimensionality reduction for expedient yield estimation of passive microwave structures. IEEE Trans. Microwave Theory Techn..

[23] Zhang Z, Cheng QS, Chen H, Jiang F (2020). An efficient hybrid sampling method for neural network-based microwave component modeling and optimization. IEEE Microwave Wireless Comp. Lett..

[24] Van Nechel E, Ferranti F, Rolain Y, Lataire J (2018). Model-driven design of microwave filters based on scalable circuit models. IEEE Trans. Microwave Theory Technol..

[25] Li Y, Xiao S, Rotaru M, Sykulski JK (2016). A dual kriging approach with improved points selection algorithm for memory efficient surrogate optimization in electromagnetics. IEEE Trans. Magn..

[26] Spina D, Ferranti F, Antonini G, Dhaene T, Knockaert L (2014). Efficient variability analysis of electromagnetic systems via polynomial chaos and model order reduction. IEEE Trans. Comp. Packaging Manufact. Technol..

[27] Zhao P, Wu K (2020). Homotopy optimization of microwave and millimeter-wave filters based on neural network model. IEEE Trans. Microwave Theory Techn..

[28] Sabbagh MAE, Bakr MH, Bandler JW (2006). Adjoint higher order sensitivities for fast full-wave optimization of microwave filters. IEEE Trans. Microwave Theory Techn..

[29] Pietrenko-Dabrowska A, Koziel S (2020). Computationally-efficient design optimization of antennas by accelerated gradient search with sensitivity and design change monitoring. IET Microwaves Ant. Prop..

[30] F. Arndt. WASP-NET®: Recent advances in fast full 3D EM CAD of waveguide feeds and aperture antennas. in *IEEE Int. Symp. Ant. Propag., APS-URSI*, Spokane, WA, pp. 2724-2727, 2011.

[31] Koziel S, Bekasiewicz A (2016). Multi-Objective Design of Antennas Using Surrogate Models.

[32] Easum JA, Nagar J, Werner PL, Werner DH (2018). Efficient multi-objective antenna optimization with tolerance analysis through the use of surrogate models. IEEE Trans. Ant. Prop..

[33] Liu B, Yang H, Lancaster MJ (2017). Global optimization of microwave filters based on a surrogate model-assisted evolutionary algorithm. IEEE Trans. Microwave Theory Techn..

[34] Cervantes-González JC, Rayas-Sánchez JE, López CA, Camacho-Pérez JR, Brito-Brito Z, Chávez-Hurtado JL (2016). Space mapping optimization of handset antennas considering EM effects of mobile phone components and human body. Int. J. RF Microwave CAE.

[35] Toktas A, Ustun D, Tekbas M (2019). Multi-objective design of multi-layer radar absorber using surrogate-based optimization. IEEE Trans. Microwave Theory Techn..

[36] Torun HM, Swaminathan M (2019). High-dimensional global optimization method for high-frequency electronic design. IEEE Trans. Microwave Theory Technol..

[37] Leifsson L, Du X, Koziel S (2020). Efficient yield estimation of multi-band patch antennas by polynomial chaos-based kriging. Int. J. Numer. Modeling.

[38] Jacobs JP (2016). Characterization by Gaussian processes of finite substrate size effects on gain patterns of microstrip antennas. IET Microwaves Ant. Prop..

[39] Zhang W, Feng F, Jin J, Zhang QJ (2021). Parallel multiphysics optimization for microwave devices exploiting neural network surrogate. IEEE Microwave Wireless Comp. Lett..

[40] Feng F, Na W, Liu W, Yan S, Zhu L, Ma J, Zhang QJ (2020). Multifeature-assisted neuro-transfer function surrogate-based EM optimization exploiting trust-region algorithms for microwave filter design. IEEE Trans. Microwave Theory Techn..

[41] Kim D, Kim M, Kim W (2020). Wafer edge yield prediction using a combined long short-term memory and feed- forward neural network model for semiconductor manufacturing. IEEE Access.

[42] Cai J, King J, Yu C, Liu J, Sun L (2018). Support vector regression-based behavioral modeling technique for RF power transistors. IEEE Microwave Wireless Comp. Lett..

[43] Petrocchi A, Kaintura A, Avolio G, Spina D, Dhaene T, Raffo A, Schreurs DMP-P (2017). Measurement uncertainty propagation in transistor model parameters via polynomial chaos expansion. IEEE Microwave Wireless Comp. Lett..

[44] Li S, Fan X, Laforge PD, Cheng QS (2020). Surrogate model-based space mapping in postfabrication bandpass filters’ tuning. IEEE Trans. Microwave Theory Tech..

[45] Koziel S, Unnsteinsson SD (2018). Expedited design closure of antennas by means of trust-region-based adaptive response scaling. IEEE Antennas Wireless Prop. Lett..

[46] Y. Su, J. Li, Z. Fan, and R. Chen. Shaping optimization of double reflector antenna based on manifold mapping. in *Int. Applied Comp. Electromagnetics Soc. Symp. (ACES)*, Suzhou, China, 1–2 (2017).

[47] Koziel S, Pietrenko-Dabrowska A (2020). On computationally-efficient reference design acquisition for reduced-cost constrained modeling and re-design of compact microwave passives. IEEE Access.

[48] A. Pietrenko-Dabrowska and S. Koziel. Numerically efficient algorithm for compact microwave device optimization with flexible sensitivity updating scheme. *Int. J. RF & Microwave CAE*. **29**(7) (2019).

[49] Koziel S, Mosler F, Reitzinger S, Thoma P (2012). Robust microwave design optimization using adjoint sensitivity and trust regions. Int. J. RF Microwave CAE.

[50] A.R. Conn, N.I.M. Gould, & P.L. Toint, *Trust Region Methods*, MPS-SIAM Series on Optimization (2000).

[51] Koziel S (2017). Objective relaxation algorithm for reliable simulation-driven size reduction of antenna structure. IEEE Ant. Wireless Prop. Lett..

[52] Tseng C, Chang C (2012). A rigorous design methodology for compact planar branch-line and rat-race couplers with asymmetrical T-structures. IEEE Trans. Microwave Theory Technol..

[53] Koziel S, Pietrenko-Dabrowska A (2019). Reduced-cost surrogate modeling of compact microwave components by two-level kriging interpolation. Eng. Opt..

[54] K. V. Phani Kumar & S. S. Karthikeyan. A novel design of rat-race coupler using defected microstrip structure and folding technique. in *IEEE Applied Electromagnetics Conf. (AEMC)*, Bhubaneswar, India, 1–2 (2013).

